# EPR Studies on the Properties of Model Photoreceptor Membranes Made of Natural and Synthetic Lipids

**DOI:** 10.1007/s12013-017-0795-4

**Published:** 2017-04-17

**Authors:** Mariusz Duda, Katarzyna Kawula, Anna Pawlak, Tadeusz Sarna, Anna Wisniewska-Becker

**Affiliations:** 1 Faculty of Biochemistry, Biophysics and Biotechnology, Jagiellonian University, Kraków, Poland; 2 Malopolska Centre of Biotechnology, Jagiellonian University, Kraków, Poland; 3 Faculty of Electrical Engineering, Automatics, Computer Science and Biomedical Engineering, AGH-University of Science and Technology, Kraków, Poland

**Keywords:** Photoreceptors, Membrane, Lipid, Zeaxanthin, Oxidation, EPR

## Abstract

The membranes of retina photoreceptors have unique lipid composition. They contain a high concentration of polyunsaturated docosahexaenoic acid, with six double bonds, and are enriched in phosphatidylethanolamines. Based on their phospholipid composition and cholesterol content, membranes of photoreceptors can be divided into three types: plasma membrane, young disks membranes, and old disks membranes. High amount of docosahexaenoic acid, abundant illumination, and high respiratory demands make these membranes sensitive to oxidative stress and lipid peroxidation. Human retinas are not easily available for research, therefore most research is done on bovine retinas. However, to follow, in a controlled manner, the changes in membrane properties caused by different factors it seems advisable to apply carefully prepared models of photoreceptor membranes. Using synthetic lipids we prepared liposome models of three types of photoreceptor membranes, and by means of electron paramagnetic resonance spectroscopy and spin labeling technique we compared polarity and fluidity of those model membranes with the properties of membranes consisting of natural lipids extracted from photoreceptor outer segments of bovine retinas. Additionally, we studied the effect of oxidation on the membrane properties in the presence and in the absence of zeaxanthin, which is an antioxidant naturally present in the human retina. The results show that there are significant differences in polarity and fluidity between all investigated membranes, which reflect differences in their lipid composition. The properties of the membranes made of natural photoreceptor outer segment lipids are most similar to the ones of the models of old disks membranes. Oxidation did not change the membrane properties significantly; however, a slight ordering effect was observed in liposomes made of natural photoreceptor outer segment lipids and in the model of old disks membranes. Zeaxanthin affected polarity and fluidity mostly in the model of old disks membranes. The results show that by careful selection and appropriate proportions of lipid mixtures, it is possible to obtain synthetic membranes of the properties similar to the natural ones.

## Introduction

Lipid and protein composition of vertebrates retinas is well known. Most of the data on lipid composition of photoreceptor outer segment (POS) membranes come from research done on bovine retinas [1–4]. For obvious reasons, much less data is available on human retinas; however, it is generally accepted that lipid composition of human and bovine retina membranes (including POS) is similar [5, 6]. The detailed measurements of the fatty acid composition of human and bovine POS membranes have been lately performed by means of liquid chromatography coupled with mass spectrometry (LC-MS) (Pawlak, unpublished data). According to the results, the fatty acid composition of POS derived from both species is very similar. The plasma membrane and the disks membranes of vertebrates retina photoreceptors have different functions in the process of visual signal transduction. Therefore, their lipid and protein composition also differs [4]. Although both membranes are rich in long-chain polyunsaturated fatty acids, such as docosahexaenoic acid (DHA), with six double bonds, DHA content in the disks membranes is much higher than in the plasma membrane reaching as much as 35 mol% [1, 2, 4, 7]. Also, the molar ratio of phosphatidylcholine (PC) to phosphatidylethanolamine (PE) is 6:1 in the plasma membrane compared with 1:1 in the disks membranes [2]. There is also a difference in the composition of saturated fatty acids—those with longer chains (such a stearoyl acid) are more abundant in the disks membranes, while those with shorter ones (e.g., myristoyl acid) in the plasma membrane [1]. Both membranes contain cholesterol [8, 9]. Nearly equimolar concentrations of unsaturated fatty acids, saturated fatty acids, and cholesterol make them very similar to the raft-forming mixtures. Therefore, raft domains (in form of detergent-resistant membranes) can be isolated from the photoreceptor membranes by cold Triton X-100 extraction [9–11]. The basic lipid composition of the disks membranes changes with the age of disks [8, 9]. Disks are membrane vesicles stacked along the length of the POS. The new disks are continually formed at the base of the POS and move up toward its apical tip where they are shed and phagocytized by the retinal pigment epithelium (RPE) cells [4]. This process takes about 12 days in vertebrates [12]. As a result, within one POS there are disks of different age. The main difference in the membrane lipid composition between young and old disks is their cholesterol content. It decreases from 30 mol% at the base of the POS to ∼5 mol% at the apical tip [8].

High amount of DHA characteristic for photoreceptor membranes, abundant illumination, and high respiratory demands of photoreceptor cells make these membranes sensitive to oxidative stress and lipid peroxidation. However, there are protective mechanisms that help to keep these membranes intact and the retina healthy. Among others, macular xanthophylls (lutein and zeaxanthin) have been proved to protect human retina against the oxidative damage and resulting diseases [13–16]. Lutein and zeaxanthin are accumulated in the Henle fiber layer composed of photoreceptor axons [17] and are present in the rod outer segments [5, 18]. Using a simple membrane model system we have shown previously that macular xanthophylls might not be uniformly distributed within the POS membranes—they are accumulated in the membrane domains rich in polyunsaturated lipids, and excluded from the domains enriched in cholesterol and saturated lipids (raft domains) [19, 20]. We have also shown that xanthophylls protect unsaturated lipids more effectively when they are present in the membranes made of raft-forming mixtures than in homogenous unsaturated membranes [21]. Based on these results it was suggested that the selective accumulation of macular xanthophylls in the most vulnerable regions of photoreceptor membranes may play an important role in enhancing their antioxidant properties and ability to prevent age-related macular diseases (such as age-related macular degeneration) [21, 22]. However, all these results were obtained for simple membrane systems that simulate the in vivo conditions only to a limited extent. Based on the data available on lipid composition of bovine and human retina membranes, in the present work we propose three detailed models of photoreceptor membranes: (1) a model of the plasma membrane consisting of dimyristoyl PC (DMPC), palmitoyl-oleoyl PC (POPC), palmitoyl-oleoyl PE (POPE), palmitoyl-docosahexaenoyl PC (PDHAPC), and cholesterol (PC:PE 6:1, 5 mol% PDHAPC, 40 mol% chol), (2) a model of the young disks membrane consisting of distearoyl PC (DSPC), POPE, PDHAPC, PDHAPE, and cholesterol (PC:PE 1:1, 35 mol% PDHAPC and 30 mol% chol), (3) a model of the old disks membrane consisting of DSPC, POPC, POPE, PDHAPC, PDHAPE, and cholesterol (PC:PE 1:1, 35 mol% PDHAPC and 5 mol% chol).

The main aim of the work was to investigate the physical properties of these model membranes in comparison with the membranes derived from natural bovine POS. Additionally, the effect of oxidation on the membrane properties was investigated. Finally, zeaxanthin was added to the model systems to check whether its presence may protect photoreceptor membrane lipids against peroxidation by changes in the membrane physical and chemical properties.

## Materials and Methods

### Chemicals and Biological Material

#### Phospholipids

1,2-dimyristoyl-*sn*-glycero-3-phosphocholine (DMPC), 1,2-distearoyl-*sn*-glycero-3-phosphocholine (DSPC), 1-palmitoyl-2-oleoyl-*sn*-glycero-3-phosphocholine (POPC), 1-palmitoyl-2-docosahexaenoyl-*sn*-glycero-3-phosphocholine (PDHAPC), 1-palmitoyl-2-oleoyl-*sn*-glycero-3-phosphoethanolamine (POPE), 1-palmitoyl-2-docosahexaenoyl-*sn*-glycero-3-phosphoethanolamine (PDHAPE), cholesterol and spin labels 1-palmitoyl-2-stearoyl-(n-doxyl)-*sn*-glycero-3-phosphocholine (n-PC, where *n* = 5, 10, or 16) were purchased from Avanti Polar Lipids (Alabaster, USA). Zeaxanthin was a generous gift from prof. G. Truscott (Keele University, UK). *Biological material*: Bovine eyeballs were obtained from the local abattoir (on the basis of permission of the local veterinary inspector (PIW 6041/4/2008) no. 26 and 221) and transported to the laboratory on ice. The following procedure of bovine retinas collection was performed at dim light at 4 °C. *Briefly*: Intact bovine eye globes were hemisected (by an incision around the *pars plana*), the anterior segments (cornea, lens, vitreous) were removed and the neural retina was gently peeled and cut off from the optical nerve.

### Isolation of POS from Bovine Retinas

Bovine POS were isolated according to the method described by Papermaster [23]. The whole isolation process was performed under dim red light at 4 °C. The excised retinas suspended in a homogenization solution (42% sucrose, 1 M NaCl, 0.1 M MgCl_2_ in 10 mM Tris-HCl (pH 7.4)) were homogenized gently on stirrer. Subsequently, the large pieces of retina were pelleted by centrifugation at 1000 × *g* for 7 min. Supernatant containing POS was layered on the top of a three-step sucrose gradient (0.84 M, 1.0 M, and 1.14 M) and ultracentrifuged at 4 °C for 1 h at 103,000 × *g* (Beckmann). The POS fraction was identified as a faint reddish band at the 1.0/1.14 M interface.

### Liposome Preparation

The membranes used in this work were a multilamellar dispersion of adequate mixtures of lipids. The membranes were prepared by the following method [24]. Briefly, chloroform solutions of lipids (containing 2.5 μmol of total lipid), zeaxanthin (5 mol%, if applicable), and spin labels (1 mol%) were mixed to attain the desired compound concentrations, chloroform was evaporated with a stream of nitrogen, and the lipid film on the bottom of the test tube was thoroughly dried under reduced pressure (about 0.1 mm Hg) for 12 h. A phosphate buffered saline (usually 0.5 mL) was added to the dried film at about 45 °C and vortexed vigorously. Then, the multilamellar liposome suspension underwent five freeze–thaw cycles, after which it was centrifuged at 14,000 × *g* for 15 min at 4 °C, and the resulting pellet was used for electron paramagnetic resonance (EPR) measurements. All preparations and measurements were performed in darkness or dim light and, where possible, under nitrogen or argon. To prepare liposomes based on POS lipids, lipids were extracted from POS fraction according to the Folch’s procedure [25].

### Liposome Oxidation

To avoid a possible antioxidant effect of nitroxides, liposomes prepared for oxidation did not contain a spin label. Liposome suspension was placed into the water bath (37 °C) and left with air access in dark for 7 days. After this period, the lipids were extracted according to Folch [25], and new liposomes with addition of 1 mol% of n-PC spin label were prepared for EPR measurements.

### EPR Measurements

5-PC, 10-PC, and 16-PC are phospholipid spin labels that have a nitroxide free radical moiety responsible for the EPR signal attached to the 5th, 10th, or 16th carbon atom in the alkyl chain, respectively. Therefore, information is obtained from different regions of the membrane. The EPR measurements were conducted with Bruker EMX spectrometer equipped with a temperature control unit (EMX ER 4141 VT). The suspension of multilamellar liposomes containing 1 mol% of n-PC spin label was placed in a gas permeable capillary (i.d. 0.7 mm) made of TPX and located inside the EPR dewar insert in the resonant cavity of the spectrometer. The sample was thoroughly deoxygenated with nitrogen gas (about 15 min), which was also used for temperature control. For polarity measurements, samples were frozen to −153 °C using liquid nitrogen vapor. For fluidity measurements, the EPR spectra were recorded at room temperature and at 37 °C.

Following parameters were obtained from the EPR spectra: 2A_z_ (z-component of the hyperfine interaction tensor of the nitroxide spin label) as a measure of polarity, S parameter as a measure of lipid order of phospholipid alkyl chains, and rotational correlation times τ_2B_ and τ_2C_ as a measure of phospholipid alkyl chains mobility in the membrane center [26–28].

## Results and Discussion

### Polarity Profiles

**Fig. 1:**
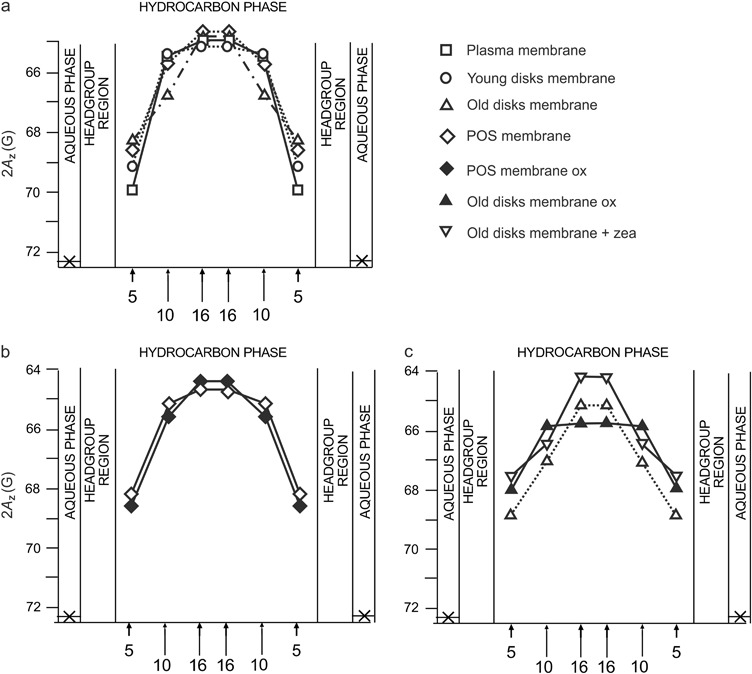
Comparison of polarity profiles across different photoreceptor model membranes: made of synthetic lipids (plasma membrane, young disks, and old disks membranes) and of natural lipids extracted from bovine POS (**a**), made of photoreceptor outer segments (POS) lipids and of oxidized POS lipids (**b**), made of synthetic lipids (old disks membranes), of oxidized synthetic lipids (old disks membranes), and of synthetic lipids (old disks membranes) in the presence of 5 mol% zeaxanthin (**c**). The spectra were acquired at −153 °C. Upward changes of 2A_z_ indicate a decrease in polarity. Approximate locations of the nitroxide moieties of spin labels are indicated by numbers under the baseline

2A_z_, which is measured directly from the EPR spectra of spin labels in frozen liposome samples as a distance between the outermost extrema, reports on the polarity of local environment of a nitroxide moiety [26, 29]. Polar solvents enhance the unpaired electron spin density at the nitrogen atom, thereby increasing the hyperfine interaction between the unpaired electron spin and the nitrogen nuclear spin. Therefore, higher values of 2A_z_ indicate higher polarity (lower hydrophobicity). Polarity profiles across the lipid bilayer reflect water penetration into the membrane [29]. This can be affected by lipid composition, presence of cholesterol, carotenoids, peptides, or drugs [24, 26, 30, 31]. Here, we analyzed the differences in polarity profiles across three models of photoreceptor membranes in comparison to the membranes formed by natural lipids extracted from POS (Fig. 1a). Also, the polarity profiles have been obtained for the POS membranes, for the old disks membranes subjected to oxidative stress, and for the old disks membranes in the presence of zeaxanthin (Fig. 1b, c). All polarity profiles presented in Fig. 1a are typical for unsaturated membranes whose hydrophobicity level in the central part is rather high [26]. The differences between the membranes in terms of 2A_z_ can be observed in the region close to the membrane surface (5th carbon) and also deeper in the membrane, around carbon 10th. This is in perfect agreement with the results presented in ref. [26] for unsaturated membranes containing cholesterol. The photoreceptor plasma membrane and the young disks membrane contain high amounts of cholesterol (40 and 30 mol%, respectively), which makes the shape of the hydrophobicity barrier in these membranes rectangular with a sharp change in 2A_z_ values between 5th and 10th carbon. Conversely, the hydrophobicity of the old disks membrane, which contains only 5 mol% cholesterol, increases gradually toward the membrane center. The values of 2A_z_ for 5-PC increase in the order: old disks < POS < young disks < plasma membrane, which reflects the increasing cholesterol content in these membranes. As shown previously, this correlates well with the depth of penetration of the steroid ring structure of cholesterol, suggesting induction of packing defects near the surface region due to the cholesterol ring structure [26]. Also, the amount of cholesterol in the model of old disks is obviously not enough to change the shape of the hydrophobicity barrier from typical for unsaturated membrane without cholesterol to that characteristic for cholesterol-containing membrane.

Oxidation of lipids does not seem to affect the polarity profiles of the POS membranes significantly (Fig. 1b). Even more surprising was the effect of oxidation in the old disks membranes where a decrease in polarity was observed at the 5th and 10th carbon positions (Fig. 1c). Since oxidation of lipids results in forming lipid hydroperoxides that are more polar than the parent compounds [32, 33] rather an increase in polarity was expected here. However, such an effect was observed only in the membrane center (for 16-PC). Jurkiewicz et al. [34], in their detailed review on the effects of oxidatively modified phospholipids on membrane biophysical properties, divided lipid oxidation products into two groups: (1) hydroxy-dieonyl or hydroperoxy-dieonyl phospatidylcholines, (2) phospatidylcholines with oxidized and truncated chains with either aldehyde or carboxylic group. Generally, the results obtained by different techniques, such as EPR, fluorescence, and molecular dynamics simulations, show that the effects of the first group of oxidized lipids on membrane properties are much weaker as those of the other group. In our experiments lipid autoxidation took place—in rather mild conditions, without any additional oxidizing agents and in the dark. LC-MS analysis of the changes in phospholipid composition of models of retina membranes subjected to autoxidation in the same conditions showed that lysolipids appeared only after 216 h of the process (Pawlak, unpublished data). Fuchs et al. [35] also suggested that longer peroxidation is necessary to obtain cleaved and fragmented alkyl chains. Therefore, it is most probable that significant modification of lipids, such as cleavage of the alkyl chains, did not take place here. Megli and Russo [36] have shown that oxidized conjugated diene species (first group of oxidized lipids) did not disorder the lipid bilayer, while most oxidized lipid molecules examined in their study were able to induce bilayer disordering. Also, the polarity profile of liposomes made of pure conjugated diene species was similar to that of normal phospholipids [34]. A weak effect of oxidation observed in our models of photoreceptor membranes may also result from a moderating influence of cholesterol [34].

Zeaxanthin clearly increases hydrophobicity in all depths of the old disks membrane that confirms the results obtained previously for simpler membrane models [24]. Generally, the high hydrophobicity barrier observed in all models of photoreceptor membranes as well as in membranes made of natural lipids extracted from POS suggests that these membranes are protected against water penetration, which should make them relatively resistant to reactive compounds dissolved in water such as charged free radicals and transient metal ions. The presence of macular xanthophylls such as zeaxanthin may additionally enhance this protective structural effect. It has to be mentioned here that zeaxanthin concentration used in our research (5 mol%) is much higher than the average xanthophyll concentration in POS, which does not exceed 1 mol% [17]. However, due to selective accumulation of zeaxanthin in the domain enriched in unsaturated lipids [19, 20], its local concentration in this domain may be higher than in the whole POS membranes.

### Membrane Fluidity—Order Parameter

**Fig. 2:**
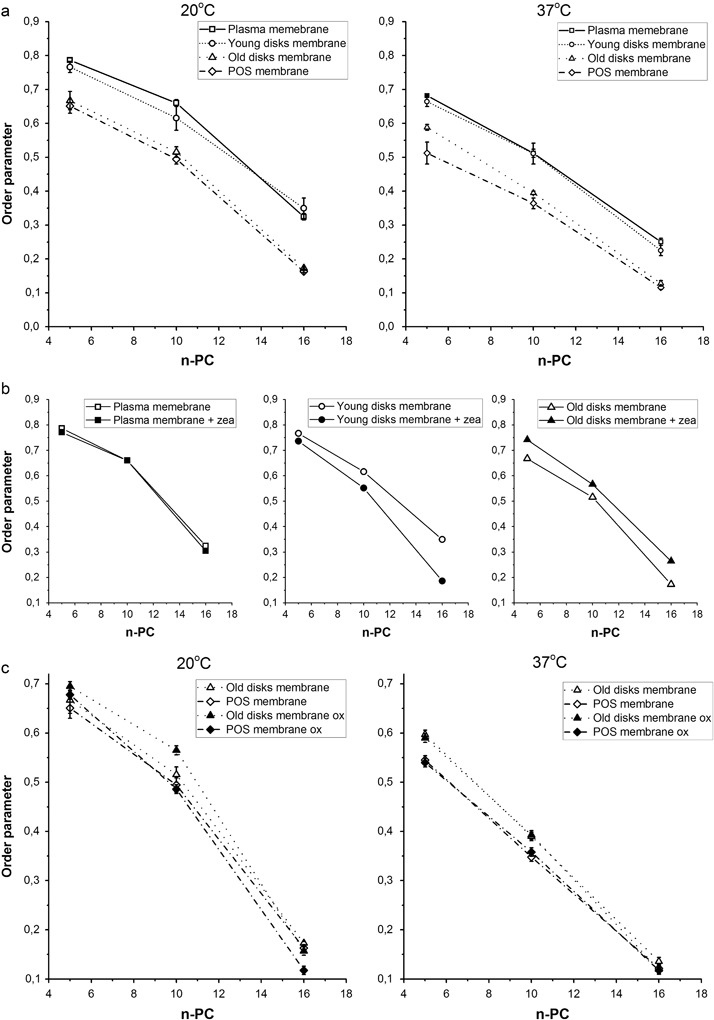
Order parameter (S) of 5-PC, 10-PC, and 16-PC in different photoreceptor model membranes: made of synthetic lipids (plasma membrane, young disks, and old disks membranes) and of natural lipids extracted from bovine POS (**a**), made of synthetic lipids (plasma membrane, young disks, and old disks membranes) in the absence and in the presence of 5 mol% zeaxanthin (**b**), made of synthetic lipids (old disks membranes) and of POS lipids subjected or not to oxidative stress (**c**). The spectra presented in **b** were acquired at 20 °C

The order of lipid alkyl chains in photoreceptor model membranes was investigated in terms of order parameter S. As expected, in all investigated membranes the values of S parameter decreased in the direction from the region close to the membrane surface toward the membrane center (Fig. 2a). At both studied temperatures (room temperature and 37 °C) the most ordered were the models of plasma membranes followed by young disks membranes, while the models of old disks membranes and POS membranes were much less ordered. As mentioned above, the plasma membrane and the young disks membrane both contain high amounts of cholesterol so the higher lipid order observed in these membranes can be attributed to its well-known rigidyfing effect [37, 38]. As far as S parameter is concerned, natural POS membranes seem to be most similar to the old disks membranes. This suggests that avarage composition of whole POS membranes resembles most the composition of old disks membranes, meaning that old disks dominate in POS. Generally, it is assumed that membranes of disks constitute approximately 90% of the total POS membranes, while the average cholesterol content of the total POS membranes is approximately 10 mol% [4]. Higher rigidity and hydrophobicity of the plasma membrane and the young disks membranes make them more resistant. Those of older disks that may be less resistant (i.e., more permeable to polar and most probably non-polar molecules as well) are successively removed by the mechanism of old disks shedding followed by their phagocytosis by RPE cells [39].

The effect of zeaxanthin on lipid order is shown in Fig. 2b. Previously, dipolar xanthophylls were proved to rigidify the membranes [40, 41]. It was even suggested that polar carotenoids may play a structural role of cholesterol in the membranes of some Procaryota that do not synthesize cholesterol [42]. However, our results in this work show the ordering effect of zeaxanthin only in the models of old disks membranes, in which the S parameter incresaes by about 0.1 at all depths, while lipid order in two other models remains unchanged or is even decreased in the presence of zeaxanthin. This can be explained by different cholesterol content of the three types of POS membranes, which results in different zeaxanthin solubility in these membranes. It has been suggested that in domain-forming membranes like POS, xanthophylls are repelled from the regions with high cholesterol content because of steric nonconformability between these two rigid molecules [43]. Also, Socaciu et al. [44] have shown that carotenoids incorporation yield into liposome membranes is significantly reduced by cholesterol. This effect was explained by the competition of cholesterol and carotenoids with respect to their incorporation into liposomes. This all suggests that old disks membranes containing less cholesterol may accommodate more zeaxanthin. To the best of our knowledge, however, there is no data available on distribution of zeaxanthin within the young and old disks.

Lipid order was also investigated in the models of POS and old disks membranes composed of oxidized lipids. In both cases no significant effect of oxidation was observed (Fig. 2c). As mentioned above, the lack of a significant effect may result from mild conditions of oxidation. Oxidized conjugated diene species, which are most probably formed during mild autoxidation, were shown previously to be unable to disorder the phospholipid bilayer in contrast to extremely oxidized cleaved chain PC which were revealed to be responsible for the observed strong EPR anisotropy loss induced by Fenton-oxidized PC [36, 45].

### Membrane Fluidity—Rotational Correlation Times

**Fig. 3:**
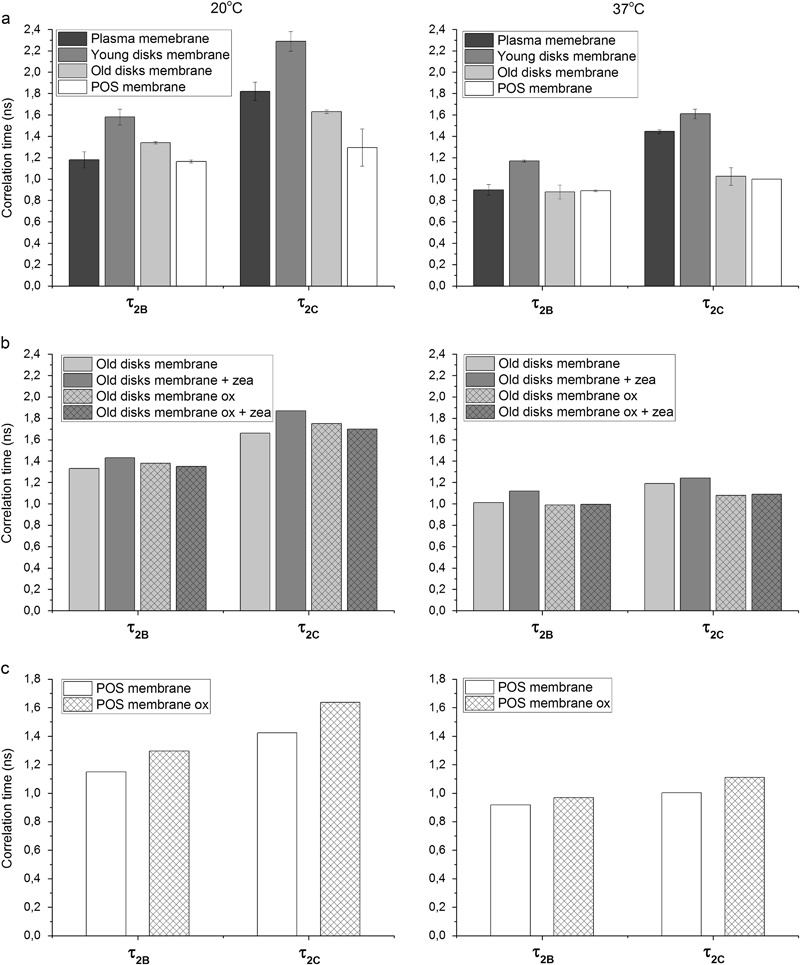
Rotational correlation times τ_2B_ and τ_2C_ of 16-PC in different models of photoreceptor model membranes: made of synthetic lipids (plasma membrane, young disks, and old disks membranes) and of natural lipids extracted from bovine POS (**a**), made of synthetic lipids (old disks membranes) in the absence and in the presence of 5 mol% zeaxanthin and subjected or not to oxidative stress (**b**), made of POS lipids and of oxidized POS lipids (**c**)

The rotational correlation times τ_2B_ and τ_2C_ of 16-PC are dynamic parameters that report on the fluidity of the membrane in its center. They were calculated using the formulas provided by Berliner [28]. The bigger the differences between τ_2B_ and τ_2C_, the more anisotropic the motion of 16-PC chains becomes. Figure 3a shows that the correlation times of 16-PC in all investigated membranes are different. The most restricted motion was observed in the model of young disks membranes, whereas the old disks membranes and especially the POS membranes were more fluid. Again, the most similar to the POS membranes were the old disks membranes, especially at 37 °C. Also, the anisotropy of the lipid motion was more pronounced in the models of plasma membranes and young disks membranes compared with the old disks and POS membranes. At 20 °C the differences between τ_2B_ and τ_2C_ in the young disks membranes and plasma membranes were 30 and 33%, respectively, whereas in the old disks and POS membranes were only 16 and 9%. At 37 °C the differences in the anisotropy of the lipid motion were even more pronounced. This again reflects the differences in cholesterol content between the investigated membranes. The higher values of correlation times in the young disks membranes compared with the plasma membrane can be explained by other than cholesterol content differences in their lipid composition: the young disks membranes contain more PE (PC:PE ratio 1:1) than the plasma membrane (PC:PE ratio 6:1). Also, the saturated PC present in the young disks membranes (DSPC) has longer chains than the main saturated PC in the plasma membrane (DMPC). Both, PE and long saturated chains make membranes more compact and their phase transition temperature is higher [46, 47]. Although our models of old disks membranes are composed of the same phospholipids as the models of young disks, they contain less cholesterol, which makes them more fluid.

As can be seen in Fig. 3b, zeaxanthin increases the rotational correlation times by only 7–10% in the model of old disks membranes; however, this observation confirms the rigidifying effect observed before with S parameter for 16-PC (Fig. 2b). Also, oxidation of lipids increases the correlation times in the models of POS membranes by 12–14% (Fig. 3c). Interestingly, although both zeaxanthin and oxidation seem to change membrane properties in the same direction, the additive effect of these two factors was not observed in the old disks membranes (Fig. 3b). Conversely, the values of correlation times in the old disks membranes containing zeaxanthin and subjected to oxidation remained the same as in the not treated membranes. It can be suggested that zeaxanthin protects lipids against peroxidation by known antioxidant mechanisms, such as free radical scavenging; therefore, the lipids retain their original properties, but at this process zeaxanthin undergoes degradation, which does not allow it to affect the membrane in a usual way. Such a degradation process was observed by Khachik et al. [48], who identified different oxidation products of zeaxanthin and lutein after 3 days of oxidation. They showed that approximately 10% of these carotenoids in both crystalline form and THF solutions underwent in-chain oxidative cleavage and degradation to give a number of carotenoid aldehydes known as apocarotenals. Such derivatives may not affect membrane physical properties. Our results are only preliminary and need to be confirmed in further experiments. Among others, we plan to check the effect of degraded zeaxanthin on the POS membrane models.

## Conclusions

The results obtained in this work show that the model membranes mimicking those of three different regions of POSs differ in terms of their physical and chemical properties. The plasma membrane seems to be the most rigid due to the highest amount of cholesterol (40 mol%), while the old disks membrane containing only 5 mol% cholesterol is the most fluid. However, unsaturation and cholesterol make all the membranes rather resistant to water penetration, and this effect may be enhanced by the presence of zeaxanthin, especially in the old disk membranes, which probably can accommodate most zeaxanthin. This may be helpful in protecting membrane lipids against an attack of hydrophilic free radicals and other reactive species. When comparing the properties of the membranes made of natural POS lipids with those of purely synthetic models, the similarity was observed between POS membranes and the old disks membranes. This suggests that the old disks prevail in POS. A slight rigidyfing effect of oxidation was observed only in the membrane center. The lack of a strong effect can be attributed to the mild conditions of autoxidation that did not result in the production of strongly modified oxidized derivatives able to affect the membrane properties significantly. In nature, however, the retina is subjected to a higher stress, since the disk membranes are oxidized up to 12 days, in the light and in the presence of intrinstic photosensitizers, such as rhodopsin photobleaching products. Additionaly, there are other factors affecting the oxidation process in vivo. For example, in the older disks there is an increase in phosphatidylserine (PS) content, with PS being exposed to the external leaflet of the lipid bilayer which gives a signal to phagocytosis. Also, there is an increasing amount of already oxidatively modified lipids and proteins that accelerate the oxidation process. Such a complex system is not possible to reproduce, but some improvement will be considered in our future experiments. Bearing in mind that the use of well-defined oxidized phospholipid species for membrane oxidative stress guarantees a more reliable and detailed response, we plan to introduce synthetic lipid hydroperoxides and/or exogeneous photosensitizers to our models. Also, we may try and differentiate more the lipid composition, and make an attempt to identify the lipid oxidation products at variuos stages of oxidation.
